# Weaknesses in the continuity of care for preterm infants discharged from the neonatal unit*

**DOI:** 10.1590/1980-220X-REEUSP-2023-0228en

**Published:** 2024-03-18

**Authors:** Mariana Camargo Tanaka, Fabiane Blanco Silva Bernardino, Patrícia Pinto Braga, Lidiane Cristina da Silva Alencastro, Maria Aparecida Munhoz Gaíva, Cláudia Silveira Viera

**Affiliations:** 1 Universidade Federal de Mato Grosso, Faculdade de Enfermagem, Cuiabá, MT, Brazil.; 2 Universidade Federal de São João del Rei, Divinópolis, MG, Brazil.; 3 Universidade Estadual do Oeste do Paraná, Faculdade de Enfermagem, Paraná, PR, Brazil.

**Keywords:** Infant, Premature, Continuity of Patient Care, Patient Discharge, Health Personnel, National Health Strategies, Recien Nacido Prematuro, Continuidad de la Atención al Paciente, Alta del Paciente, Personal de Salud, Estrategias de Salud Nacionales, Recém-Nascido Prematuro, Continuidade da Assistência ao Paciente, Alta do Paciente, Pessoal de Saúde, Estratégias de Saúde Nacionais

## Abstract

**Objective:**

To identify weaknesses in the continuity of care for preterm infants discharged from a neonatal unit, based on the perspective of professionals in the family health strategy.

**Method:**

Qualitative research, carried out with 16 professionals from four health regions in a capital city in the center-west of Brazil. Data collection took place from October to December 2020, through semi-structured, individual, and in-person interviews. Data underwent content analysis, supported by the concept of continuity of care.

**Results:**

The analysis consisted of three categories: Challenges for care in the unit and referral to specialized services; weak interactions between the preterm baby’s family and health professionals; Information: essential aspect for the connection between health professionals and the family of the preterm newborn.

**Conclusion:**

Health services are shown to be fragile in terms of the dimensions of continuity of care, contributing to the discontinuity of care for preterm children.

## RESUMO

**Objetivo:**

Identificar as fragilidades para a continuidade do cuidado ao pré-termo egresso de unidade neonatal, a partir da perspectiva de profissionais da estratégia saúde da família.

**Método:**

Pesquisa qualitativa, realizada junto a 16 profissionais de quatro regionais de saúde de uma capital do centro-oeste do Brasil. A coleta dos dados ocorreu nos meses de outubro a dezembro de 2020, por meio de entrevistas semiestruturadas, individuais e presenciais. Os dados foram submetidos à analise de conteúdo, sustentada pelo conceito da continuidade do cuidado.

**Resultados:**

Três categorias compuseram a análise: Desafios para o atendimento na unidade e para o encaminhamento aos serviços especializados; Interações frágeis entre família do pré-termo e profissionais de saúde; Informação: aspecto essencial para a conexão entre profissionais de saúde e família do recém-nascido pré-termo.

**Conclusão:**

Os serviços de saúde mostram-se frágeis quanto às dimensões da continuidade do cuidado colaborando para a descontinuidade da atenção à criança nascida pré-termo.

## RESUMEN

**Objetivo:**

Identificar debilidades en la continuidad de la atención al recién nacido prematuro egresado de una unidad neonatal, desde la perspectiva de los profesionales de la estrategia de salud de la familia.

**Método:**

Investigación cualitativa, realizada con 16 profesionales de cuatro regiones sanitarias de una capital del centro-oeste de Brasil. La recolección de datos se realizó de octubre a diciembre de 2020, mediante entrevistas semiestructuradas, individuales y presenciales. Los datos fueron sometidos a análisis de contenido, sustentado en el concepto de continuidad de la atención.

**Resultados:**

Tres categorías comprendieron el análisis: Desafíos para la atención en la unidad y derivación a servicios especializados; Interacciones débiles entre la familia del bebé prematuro y los profesionales de la salud; Información: aspecto esencial para la vinculación entre los profesionales de la salud y la familia del recién nacido prematuro.

**Conclusión:**

Los servicios de salud son frágiles en términos de continuidad de la atención, lo que contribuye a la discontinuidad de la atención a los niños nacidos prematuros.

## INTRODUCTION

Technological advances in the diagnosis and management of newborns who require neonatal hospitalization have increased the chances of survival for this age group. It is also known that the adequate development of these children is determined by a balance between meeting their biological, environmental and family needs^(1)^. The challenges faced by families begin with the birth of a preterm child, as they begin to seek the best care and monitoring, both during hospitalization and after discharge. Along the way, they encounter access difficulties or lack of services, which can be challenging^(2)^.

Based on the need to qualify and humanize the care provided to Pre-Term (PTNB) and/or Low Weight (LW) Newborns and their families, in the Brazilian scenario, the Ministry of Health (MS) created, in 1999, the public policy “Standard for Humanized Care for Low Weight Newborns (NAHRNBP) – Kangaroo Care (*MC*)”.

Monitoring of PTNBs after discharge from the Neonatal Intensive Care Unit (NICU) generally takes place in follow-up outpatient clinics linked to the hospitals where they were hospitalized and concomitantly in primary health care (PHC). Monitoring children born preterm in PHC is one of the actions proposed by the National Policy for Comprehensive Child Health Care (PNAISC), established in 2015 by the Brazilian Ministry of Health, which aims to ensure comprehensive, resolute, and humanized care aimed at reducing child morbidity and mortality, using the Manual for the Third Stage of the Kangaroo Method in Primary Care as a guide^(3)^.

In primary care, child monitoring must be based on health promotion, which is closely related to health surveillance and child development, whose actions are capable of reducing situations of vulnerability and risks, favoring early identification and intervention faced with developmental delay and growth complications in childhood^(3)^.

Therefore, understanding how the continuity of care for preterm infants occurs after hospital discharge requires an accurate look that sees continuity as a series of health care events experienced by users as coherent and connected care, which considers, in addition to clinical needs, the personal context of each individual and lasts over time, and is operationalized in three dimensions: informational continuity, management continuity, and relational continuity, which can be identified in different health care contexts^(4)^.

Continuity of care is favored through the personal relationship between patient and professional, the communication of relevant information, and the cooperation among professionals within and between health services^(5)^. Therefore, it is a complex and multifaceted process that requires adequate communication, knowledge, experience, competence, and skills from the team^(6)^.

In developed countries, especially the United States of America and some European countries, the search for continuity of care is ensured by the transition of care and driven to the improvement of care quality and life of users, reducing costs with avoidable readmissions^(7)^. However, in Brazil and in border countries such as Paraguay and Argentina, the literature reveals deficiencies in coordination between different levels of care, resulting in difficulties in accessing health services^(6,8,9)^.

Weaknesses in the continuity of care can contribute to fragmented and isolated care, not meeting the demands of this vulnerable population coming from the NICU^(6,10)^. To understand continuity of care and translate this understanding into policies, it is essential to look at the concept within the general context of health services, with emphasis on access and quality of care^(5)^, especially knowing the perspective of the subjects who provide care in primary care.

Brazilian studies have been exploring the theme of continuity of care for preterm infants; however, indicators show that we still live with low adherence to follow-up and high dropout rates^(9)^. Allied to this, we identified that despite the relevance of the theme and the wide dissemination in the literature on the conceptual and practical exploration of continuity of care, with themes focused on children’s health and chronic conditions^(11)^, in the hospital setting^(12)^, and on the understanding of primary health care nurses regarding the concept^(7)^, a gap is still evident, based on the perception of primary care professionals regarding the continuity of care for preterm infants discharged from the NICU. Involving all primary care professionals is important because the continuity of health care is achieved through interpersonal relationships, information sharing, and effective coordination of interventions, with these elements being attributes of PHC that must qualify the Health Care Network (*RAS*)^(12)^.

Given the above, the following question arises: How do health professionals from the family health strategy perceive the difficulties in continuing care for preterm infants discharged from the NICU? Therefore, this study aimed to identify weaknesses in the continuity of care for preterm infants discharged from a neonatal unit, based on the perspective of professionals in the family health strategy.

## METHOD

### Design of Study

This is a descriptive, exploratory study with a qualitative approach, supported by the concept of continuity of care^(4)^. This concept is discussed based on three dimensions: informational continuity, management continuity, and relational continuity, which can be identified in different health care contexts. Informational continuity refers to aspects related to information and represents the essential element for connecting the care of different professionals and an individual’s health event. The dimension related to management continuity refers to the management of care provided by different professionals, allowing action with shared objectives. The dimension of relational continuity, on its turn, corresponds to the establishment of continuous relationships between professionals and users^(4)^.

The tool *Consolidated criteria for REporting Qualitative research* (COREQ), Portuguese version^(13)^ was used to guide the design and presentation of research results.

### Population, Local and Selection Criteria

The study was developed with teams from the Family Health Strategy (FHS) in a capital city in the center-west of Brazil that currently has 87 primary health units, 21 of which are traditional Health Centers and 66 are Family Health Strategy Units, distributed in four health regions: East, West, North and South. For this research, the units to be studied were chosen through a draw, including one unit from each macro-region, totaling four services.

The participants were workers from different professional categories - Physician, Nurse, Nursing Technician, and Community Health Agent (*ACS*) working in the drawn FHS teams, totaling 16 professionals.

The following inclusion criteria were adopted: having been in the position for at least six months, being a public servant or hired by the Municipal Health Department and whose team consisted of at least one physician, one nurse, one nursing technician, and one *ACS*. The second team drawn for interview was replaced, since it was incomplete and a new draw was carried out, and the interview was directed to another team that met all the criteria. Professionals away from their work activities or on vacation during the data collection period were excluded from the research.

The number of participants was considered sufficient, as they generated recurring data and complementary information, thus reflecting the multiple dimensions of the study phenomenon in intensity and depth, and with the purpose of enhancing the research and making it defensible^(14)^.

### Data Collection

Fieldwork took place between October and December 2020, through individualized interviews, guided by a semi-structured script consisting of sociodemographic data and the following guiding questions prepared by the researchers: How do you establish communication with other health care services when assisting preterm children discharged from the NICU? How is preterm health information managed in the unit? Is there a unified monitoring plan developed for preterm babies and families in the Health Care Network? How do you describe the relationship established with the families of preterm children?

The professionals were contacted via messaging application to schedule the interview. Since services to users were suspended in the health units, the interviews were carried out in one of the offices available in the units and lasted an average of 15 to 20 minutes, being audio recorded with the consent of the participants. As data collection took place amid the SARSCoV-2 pandemic, biosafety and contamination prevention measures were followed by the researcher and interviewees.

### Data Analysis and Treatment

Data were organized and subjected to content analysis in the thematic modality following the steps^(14)^: comprehensive and exhaustive reading; exploration of the material; processing and interpretation of data. First, each of the interviews was read, seeking to identify, in the professionals’ speech, the elements indicative of their perceptions about the phenomenon under study. Next, similarities and divergences in the content expressed in the speeches were sought, which were grouped into broader categories, according to the research objectives. To ensure the reliability of the data, it was transcribed by the main researcher and reviewed by the second researcher. In addition, double coding and checking for discrepancies were carried out to ensure the validity of the construction of the code tree.

Next, data were interpreted, taking as reference the concepts of the dimensions of continuity of care^(4)^. This theoretical structure allowed the establishment of links between the ways professionals perceive continuity of care for preterm infants discharged from a neonatal unit.

### Ethical Aspects

The study was approved by the Research Ethics Committee, with consolidated opinion number 2.788.928 of 2018. The research complies with Resolution 466, of December 12, 2012. To guarantee anonymity, the letter “E” was used, which means “Interview” (Entrevista in Portuguese), to identify the study participants, followed by an Arabic number (E1, E2, E3, E4, E5, E6, E7, E8), according to the chronological order in which the interviews took place and, finally, the initial of position of the interviewed professional, that is, if it is “Nurse”, the letter E (for Enfermeiro, in Portuguese), if “Physicianr”, the letter M (for Médico in Portuguese), if “Nursing Technician” the letter T, if Community Health Agent “A; therefore, each speech report will be presented with the code described [E1E], [E2M], [E1T], [E3A].

## RESULTS

Sixteen professionals participated in the study, all women, aged between 26 and 59 years old, including four physicians, four nurses, four nursing technicians, and four *ACS*s. Data analysis allowed us to reveal the weaknesses present in the managerial, relational, and informational dimensions that interfere with the continuity of care for preterm infants discharged from the NICU and their families. Thus, the data was categorized into three themes: Challenges in caring for preterm infants in the unit and referring them to specialized services; Informational weaknesses in the preterm care network and weak interactions between the preterm’s family and professionals (Chart 1).

**Chart 1:** Themes generated from the interviewees’ statements – Central-West Region, Brazil, 2020.

Dimension	Evidence	Examples from Interviews	Theme
Managerial	The analysis pointed to difficulties in referring preterm infants to specialized services and prolonged time to carry out exams, in addition to the inefficiency of referral and counter-referral, which indicate the need for improvements in written and verbal communication between professionals from different points of the care network.	*What makes it difficult is the lack of tools themselves. Vacancy for baby hearing test and tongue-tie assessment. This is more complicated, it takes too long, the heel prick test also takes too long* (E2A). *Our hands are tied, because there are some referrals that take time to obtain. There are things you can’t solve. This is not part of primary care itself, it is a problem with the SUS* (E2M). *What makes it difficult is the delay in getting places in referrals for certain specialties. Because I think there are very few neuropediatricians, for example, in Cuiabá* (E4A). *You refer this child or mother to the reference service at the time of birth and, generally, the reference service -when they are teaching hospitals - tends to be a little better, everything is explained in details, while others are not so good. The existing communication is the written reference and counter-reference. I think it could still improve* (E1M).	Challenges in caring for preterm infants in the unit and referring them to specialized services
Managerial	Professionals indicate difficulties regarding access and availability of inputs, equipment, and work instruments in their units.	*The difficulties are related to the network, I think the network is a little weakened as a whole, not only regarding premature babies and equipment management... unfortunately I have been asking for more than a year and I think the issue of the anthropometric tape used to take measurements is on a bidding process. We improvised a tape, because we don’t have any* (E1E). *There is lack of material, there is no otoscope, so it is an amount that I cannot afford to leave in the unit. We have already placed an order, the health department takes forever to respond, so far, nothing* (E2M). *A premature child in our area needed a gastrostomy. The procedure requires materials and costs a lot, but the bottles and equipment don’t come to primary care, so we depend on another level of care. We guide the mother to obtain the materials in other places* (E1E).	
Relational Management	Data analysis showed difficulties or delays in referring preterm infants to specialties or other RAS services, in urgent situations. Situations were also identified in which scheduling is only made possible due to the informal contact that the FHS professional makes, to try to resolve the demand.	*Some referrals take us a while to book. Sometimes if we have someone we know in a hospital to try to speed things up, we try, but this is through the doctor, the nurse, if there is someone we know who can help, you know. But via SUS, the problem sometimes takes a long time* (E2M). *This communication is established via SISREG (National Regulation System), but they try to achieve it in another way. A phone call to someone you know, something more informal* (E3M). *If we need another service, in this case we understand that as the child is premature or even other types of special cases, for example “I need a pediatrician now”, I will not send this child to the Regulation Center and wait, you know - we don’t know how many days to get a consultation. So we call our colleagues “You see, I have a pediatrician at Health Center X”, so we call, book the consultation* (E3E).	
Relational Informational	The content of the interviews made it clear that professionals from the FHS teams are not sufficiently clear about their role in monitoring children and that they may be unaware of the role of neonatology outpatient clinics/follow-up.	*This last child who has hydrocephalus came in November and I went to her house. Then in December the child was hospitalized and the health agent monitored her, admitted her again, and now she’s back from the hospital, I go to her house, but the mother is staying more there (at the Neonatology outpatient clinic) than here. It often happens that they spend more time at the clinic than here. The first year, I understand, but there are many mothers who come with a four-year-old child who was born pre-term and want to continue monitoring at the outpatient clinic. It’s something that is no longer needed* (E2E). *They usually come with a letter to follow up. When this is not the case, they already make a referral there to enter their own premature clinic. And after they are discharged from the outpatient clinic, they come with a letter for us to follow up with childcare* (E4M).	Informational Fragilities in the preterm care network
Informational	The lack of communication with the hospital where the preterm baby was admitted is a reality of the investigated scenario. Despite obtaining information about the birth and the postpartum woman’s health status, mother of the PTNB, the professionals claim that they do not receive enough information about the premature baby’s clinical condition.	*And another challenge, another experience with a preterm child, we don’t receive anything from the hospital, we receive the information that the mother brings and the papers that the mother brings from the hospital. If it is an organized family, who has everything from the hospital, then you know everything* (E2E). *From the university hospital I receive the patient’s discharge, the mother, but I don’t receive anything from the preterm baby. I get what comes with the family, which actually belongs to the family* (E2E).	
Informational	Despite recognizing that there are specificities for the different services that care for preterm infants, it was evident that some FHS professionals are not aware of the MS guidelines in relation to monitoring preterm infants after hospital discharge.	*I think this (FHS) is an assistance that complements the assistance provided by the neonatology outpatient clinic, you know, that generally these children who are premature, they are already guided and even scheduled for consultations in the neonatology outpatient clinics of the hospital where they were born. But it is a situation that we complement in the unit, we see this patient within the community* (E1M).	
Informational	The COVID 19 pandemic hindered information management among professionals on the same team in the FHS due to the suspension of weekly multidisciplinary meetings.	*Now with the pandemic our routine has changed a little, but before we had frequent meetings to discuss cases of premature children and we looked for a solution as a team, but now it has been suspended because our focus is COVID-19* (E4E). *Generally, before the pandemic, on Friday we meet for a home visit, the health agent gives the information and then we discuss the case and make a referral, either to carry out the visit or book a consultation.* (E2E).	
Relational	Difficulties in establishing a bond with the preterm baby’s family, among which the high turnover of team professionals stands out, which reflects the poor relationship between the ACS and the community.	*We usually find out whether it is preterm or not when the mother comes to the unit looking for the first vaccination. We do not yet have, in this unit, the connection with the ACS for him-her to pass this information on to us. So we already register the child, carry out home visits, and follow-up at the unit* (E3E). *Sometimes it is difficult to maintain the entire schedule of women in prenatal care and also of those who are in the postpartum period, in addition to all the other situations that we also have to accommodate within the family health unit. We have difficulty maintaining a team to embrace the patient from the reception desk to the physician. We have high turnover in family health teams* (E1M).	Fragile interactions between the preterm baby’s family and professionals
Relational	The large number of patients per area assigned to FHS care contributes to increased demand, weakening the bond between the community and the health unit.	*Due to the large number of patients, we are often unable to have this bond. As there are units that serve uncovered areas, the flow is very large in some units. It’s intense here too. There are premature children that I cannot give the necessary attention to, I take care of a lot of children and there has to be that bond* (E3M).	
Relational	The active search carried out by the ACS for the family to have follow-up at the FHS is a reality identified in the research scenario. In the perception of professionals, the family may not recognize the importance of the follow-up carried out by the FHS.	*The right monitoring would have to be one hundred percent, but if it isn’t, we have to go for it and bring up the problem, it ends up being very difficult for the mother to come. As she already has greater support, which is the hospital, they think that the support from primary care is very little.* (E4A).	

Source: Survey data.

Data analysis showed that the continuity of care for PTNBs and their families was considered by the professionals interviewed as fragile in its managerial, relational, and informational dimensions, due to different aspects presented by them that lead to challenges to be faced in the care of those leaving the Neonatal ICU in PHC.

## DISCUSSION

With the present study, it was identified that the continuity of care for PTNBs discharged from a neonatal unit presents weaknesses that interfere with its effectiveness, leading to challenges that have to be faced. These weaknesses involve the management dimension, identified by the inefficient aspects of referral and counter-referral, lack of basic material for care, slow access to RAS services. To this end, it is identified that longitudinality for care, which is one of the essential pillars of PHC, is impaired. This attribute comprises the continuity of care over time, mediated by relationships of bond and trust between the user and the health professional, which proved to be weakened in the present study. Based on longitudinality, it is possible to know the users and their context, as well as their behaviors, habits, and health problems, to propose appropriate care and interventions^(15)^.

Furthermore, fragility was observed in the attribute of comprehensiveness for care in PHC, since professionals reported difficulties in caring for preterm infants in the FHS and their referrals to specialized services, hindering resoluteness of care, a key element in the attribute of comprehensiveness.

Professionals reported difficulties in accessing specialties, lack of basic supplies for care, fragile referral and counter-referral. In this context, professionals end up having to use informal tools to be able to refer PTNBs.

Therefore, it is clear that the continuity of management in these services is insufficient to guarantee the quality of care for preterm infants discharged from the neonatal unit. This is because care does not occur in a complementary and timely manner, aiming at meeting the common objectives of care and attention for PTNBs, which hinders the users’ access to health services and does not allow for flexibility and adaptation of the care provided over a long period of time.

The results of the present investigation are in line with another study that showed that the dimension related to continuity of management in the follow-up of preterm babies is still deficient and there is a need for articulation between the different levels of care and the construction of a line of care in the assistance provided to children discharged from the NICU in the third stage of *MC*. However, professionals do not know how to put it into practice^(12)^, which predisposes the discontinuity of follow-up of the child discharged from the NICU. This lack of articulation between the different levels of care has been a challenge for years, in several other areas of health care, where there is a loss in the continuity of care to be provided, due to the difficulty in recognizing the responsibilities of each professional in the articulation of RAS services^(16)^.

Furthermore, the articulation between the primary and tertiary health levels is still fragile and does not seem to be present in the daily life of the FHS, with PTNB and/or low birth weight baby monitoring being carried out almost predominantly in hospital follow-up outpatient clinics. An issue that seems to contribute to the non-appreciation of counter-referral for FHS professionals is that although they recognize the importance of these units in the continuity of care, they find themselves unprepared to follow the PTNB and/or LBW baby, highlighting the need to receive adequate training to deal with these children’s peculiarities^(17)^. It is then observed that continuing education in PHC, even though it is a national health policy and strategy of the Brazilian Public Health System (*SUS*), is not a reality in the locations investigated. The qualification of health professionals can contribute to the transformation of health practices in the care of PTNBs and their families, and continuing education can occur through the use of technologies such as the internet, distance education, and telehealth^(18)^.

It should also be noted that the referral and counter-referral process in the Brazilian health system is still deficient and communication between tertiary care professionals and the FHS takes place discretely, with information being conveyed almost exclusively by the user and/or or their caregivers^(6)^. In this context, the need to develop referral and counter-referral flows for the PTNB is identified, with a view to ensuring that the PTNB and their family have access to the different RAS care points so that comprehensive care can take place^(9)^.

Furthermore, PHC coordination proved to be unsatisfactory, due to failures in communication between network services and the PHC team. Limitations in communication between PTNB families and health professionals are also present in an international context, revealing that there is no cooperation between them and identifying different expectations and lack of communication between NICU nurses and nurses from other services regarding discharge planning for these children^(19)^.

Regarding communication with the child’s hospital of origin, the professionals in the present study reported that only University Hospitals usually send discharge letters for the newborn to the FHS, but that in most cases, they are unaware of the outcomes of the hospitalization and they are dependent on the information provided by mothers to initiate appropriate monitoring.

FHS professionals state that the formal communication of referral and counter-referral of the child’s follow-up, when it occurs, is based on the information recorded in the Child Health Record (CSC) or in the discharge summary. However, there was no indication of the child’s reference Health Unit, and parents and caregivers assumed the search for care in primary care and often ended up wandering through the health system, showing a still fragmented flow^(10)^. It is reiterated that although the *CSC* is considered an important communication tool among health professionals, it was not mentioned by the participants in this study.

With regard to the referral and counter-referral process in the case of PTNBs, the *MC* strategy seeks to promote the integration of services in the RAS to provide more efficient assistance in different areas of care. From this perspective, it is essential to highlight that the points of care must function as networks and be connected with a view to minimizing the fragmentation of care and ensuring comprehensive care^(20)^. In the reality studied, a difficulty was identified in articulating the RAS, in communicating with other services, as well as little knowledge of professionals about *MC*. As in other studies, in relation to *MC*, it is evident in the speeches that some mothers and professionals recognize the benefits of the method, but reduce the process to just the kangaroo position^(21)^.

Therefore, it would be important for the institution’s managers to be aware of the implementation of good practices in neonatal health, aiming at the quality of care provided, the training of the professionals involved and providing appropriate conditions for carrying out the work developed and humanized care for the NB and their family members^(22)^.

It is understood that the managing role of the nurse is an important part in articulating with other levels of the health care network and in the internal management of care for the population described. For positive communication to occur in PHC, it is essential that there is adequate communication between levels of care so that referrals can be made and users can return to the FHS with the clinical information necessary for continuity of care^(23)^.

Other authors corroborate the findings of the present study and indicate weaknesses between the coordination of the hospital service and the FHS, in the third stage of the MC, as well as in the dependence on the monitoring of families of preterm babies in the hospital outpatient clinic to the detriment of this follow-up in primary attention^(24)^. This preference on the part of families was also mentioned by the professionals in this research, who state that the mothers themselves mentioned that, in their opinion, as it is the hospital where the child was born, monitoring in this place is more important and there is no need to add another place.

This idea by mothers is reinforced by another study that analyzed the quality of the hospital/home transition and its relationship with hospital readmissions of at-risk children. The results showed the lack of coordination between tertiary care and PHC professionals, the absence of referral and counter-referral at different points in the RAS, and the low rate of children followed up by PHC after hospital discharge^(25)^.

Continuity in its relational dimension corresponds to both a link between current care and future care, in a way that provides the individuals assisted with a feeling of predictability and coherence in care^(4)^. Thus, in the present study, it was possible to see that this dimension is closely linked to the success in the monitoring of preterm babies by the FHS. Study participants reported that when there is no bond established between the family and the team, there is little parental adherence to monitoring by the FHS. After hospital discharge, the family plays an important role in promoting care, to ensure the survival and growth and development of the preterm newborn, guided by the information received in the hospital and that provided during the initial home visit (HV) carried out by the *ACS* and other FHS professionals.

In this context, the empowerment of parental caregivers is favored by the information provided to them and the monitoring of health professionals, with their availability and listening being a determining factor in the development of the family’s leading role in preterm baby care^(26)^. As stated by the professionals interviewed in this research, the bond previously created between the family nucleus and the FHS plays an important role in all family’s needs for care, including the birth of a child at risk and their arrival in the community.

Guidance to parents/guardians on the care of preterm infants after hospital discharge must occur throughout the hospitalization process^(3)^. It is recognized that such guidelines are crucial to favor the continuity of *MC* in primary and tertiary care. In their turn, health professionals who work in maternity hospitals refer the continuity of preterm care only to the outpatient service, suppressing the sharing of care with the FHS^(21)^.

It is not expected that the FHS teams will take over the care of the specialized team, but that they will continue to carry out the work of supporting health and encouraging and facilitating the child’s stay in the different services, reinforcing the partnership between the two levels of care^(3)^.

Finally, in relation to informational continuity, this dimension proved to be underdeveloped in this study, and this reflects on the quality of the bond, as well as in the adherence to preterm monitoring, considering that the relational and informational dimensions, if not present in their completeness, impair the continuity of care after discharge, both in this research and in studies of the same nature already carried out^(27,28)^.

Professionals from different services need to have access to comprehensive information about previous care and health conditions of the individual they are monitoring, to support the implementation of care. Therefore, through information it is possible to provide coordinated assistance among different professionals and implement a care plan consistent with the user’s needs, and the user is able to continue their own care when they have the information shared^(29)^.

Regarding access to information, the lack of data on the postpartum woman and her child after birth hinders the continuity of care by the FHS. If mothers do not receive HV from the family health team, the professionals will not have access to information about patients^(21)^. Therefore, it is clear that the lack of communication, that is, the fragility in the informational dimension, on both sides, jeopardizes rapprochement of individuals and, consequently, the development of the other dimensions that constitute the continuity of care.

In this regard, it is highlighted that during the hospital-home transition period, it is essential that the child and their family are monitored early and continuously and, in addition, home visits must be carried out by ESF professionals, as well as other actions that facilitate the process of family adaptation during this period. In the present study, through the professionals’ reports, it was possible to observe that families previously informed about the need for monitoring, both by family health professionals and by the hospital team, are those most likely to create a bond with the ESF and seek monitoring of the preterm.

Some professionals interviewed in this research mention that without information being passed on by families/parental caregivers, management becomes more difficult for the service. Furthermore, many mothers prefer to monitor their children in neonatology outpatient clinics, with pediatricians and other specialists, believing that primary care does not offer the necessary assistance for the child. This situation corroborates a study showing that FHS professionals, when referring to children born preterm and/or with low birth weight, relativized care that they recognized as their responsibility, such as weight and height monitoring, vaccination, dietary guidance and hygiene care, demonstrating difficulties in recognizing children at risk as the responsibility of the entire health service network^(17)^.

A study on the quality of the hospital/home transition service, or preparation for discharge of preterm newborns, identified that in hospitals where there was an individualized discharge protocol from hospitalization, there was a reduction in hospital readmissions, which occur due to poor communication among professionals, the patient, and the family regarding guidance and home care^(30)^.

It is concluded that the follow-up of preterm children remains unknown to FHS professionals. There are difficulties identified in several studies^(2,9,10)^, which for years have mentioned some obstacles to comprehensive and longitudinal care in the follow-up of PTNBs in the FHS, among them, difficulties for teams in: coordinating and articulating the care network; implementing national protocols and guidelines; managing everyday issues and family dynamics; establishing and carrying out longitudinal therapeutic plans; as well as the need to articulate means of communication with other services in the care network. In other words, the dimensions of continuity of care are used as tools for implementing preterm care in the FHS, but they still need to be better developed, as the absence or fragility of just one of them prevents the success of the others^(4)^.

This study showed that the COVID-19 pandemic presented additional challenges to the informational dimension of continuity of care and research indicates that there are negative repercussions of this period on child care. It is worth highlighting that the pandemic context brought sanitary measures that restricted service. Thus, we infer that new studies will be developed and will be able to show the outcomes of the pandemic, in the medium and long term, on the continuity of care for preterm infants.

Among the limitations of the study is data collection during the SARS CoV-2 virus pandemic in which the FHS teams were destined to treat mild cases of the disease and the monitoring of other follow-ups were temporarily suspended. In addition, the investigation was restricted to analyzing the perception of health professionals from family health units without including professionals who work in hospitals and follow-up clinics. However, the need to deepen the approach to the procedurality involved in the continuity of care for preterm infants and each of the aspects encountered, considering the specificities of the different levels of care, is recognized.

As for the improvement provided by this research for nursing practice, it is shown that care after hospital discharge is crucial in the process for maintaining the health of children born under risk conditions and that assistance aiming at comprehensive home care is required. Therefore, nurses have a prominent role in the FHS due to their generalist training, ease of communication with other areas, experience in planning, executing, and evaluating actions. This way, they can assist in the management of information among different levels of health care and, consequently, in the continuity of care to be given to preterm babies and their families.

Therefore, the present study advances by addressing the continuity of care for PTNBs with a focus on the elements that negatively impact the continuity of this care in PHC units. In this context, the results highlighted the main difficulties, weaknesses, and challenges that have to be overcome, which could support actions to improve nursing care for PTNBs leaving the NICU. Thus, based on the findings presented, it is possible to discuss the expansion of health practices in FHS units, which includes improvements in the managerial, informational, and relational dimensions of care for PTNBs discharged from the NICU, specifically in PHC.

## CONCLUSION

The managerial challenges in the continuity of care in the investigated scenario are expressed as difficulties in relation to information management inside and outside the FHS, difficulty in family adherence to follow-up beyond the neonatology outpatient clinic and difficulty in recognizing the role of the FHS in the follow-up process of the preterm, both by the family and the health team itself.

Data showed that the relational dimension in preterm care is marked by difficulties in establishing the link between health care professionals and the family, due to the family’s lack of knowledge about the need to monitor the child both at the birth hospital’s outpatient clinic and the FHS. Consequently, this lack of knowledge/guidance also reflects the lack of a prior bond, which could be established previously or during hospitalization and preparation for discharge.

In post-discharge care for preterm infants, informational weaknesses are the sum of difficulties in establishing communication and lack of adequate guidance, contributing to the difficulties found in other dimensions, resulting in failed care management and interrupted/weakened bonds.

In the context of this study, there is, on the part of the professionals interviewed, recognition of the need to train and equip themselves with knowledge to better serve preterm infants discharged from the NICU and their families, and mainly the desire to offer more qualified assistance to the this population.
